# The Potential of Metabolomics in Colorectal Cancer Prognosis

**DOI:** 10.3390/metabo14120708

**Published:** 2024-12-15

**Authors:** Chengqu Fu, Xinyi Liu, Le Wang, Dong Hang

**Affiliations:** 1 Department of Epidemiology, Jiangsu Key Lab of Cancer Biomarkers, Prevention and Treatment, Collaborative, Innovation Center for Cancer Personalized Medicine, School of Public Health, Nanjing Medical University, Nanjing 211166, China; fuchengqu@stu.njmu.edu.cn (C.F.); xinyiliu@stu.njmu.edu.cn (X.L.); 2 Department of Cancer Prevention, Zhejiang Cancer Hospital, Hangzhou Institute of Medicine (HIM), Chinese Academy of Sciences, Hangzhou 310022, China; wangle@zjcc.org.cn; 3 Changzhou Medical Center, Nanjing Medical University, Changzhou 213000, China

**Keywords:** colorectal cancer, biomarker, metabolomics, prognosis

## Abstract

Colorectal cancer (CRC) is one of the most common cancers worldwide, posing a serious threat to human health. Metabolic reprogramming represents a critical feature in the process of tumor development and progression, encompassing alterations in sugar metabolism, lipid metabolism, amino acid metabolism, and other pathways. Metabolites hold promise as innovative prognostic biomarkers for cancer patients, which is crucial for targeted follow-up care and interventions. This review aims to provide an overview of the progress in research on metabolic biomarkers for predicting the prognosis of CRC. We also discuss the future trends and challenges in this area.

## 1. Introduction

Colorectal cancer (CRC) ranks as the second leading cause of cancer-related mortality worldwide [1]. Despite continual advancements in treatment modalities, such as surgery, chemotherapy, and radiation therapy, the overall mortality rate among CRC patients remains notably high [2]. Due to the insidious onset of CRC, approximately 65% of patients are diagnosed at an advanced stage, with a five-year survival rate of less than 15% [3]. Accurately predicting patient survival and implementing precision interventions have been a longstanding research focus. Currently, the American Joint Committee on Cancer (AJCC) TNM staging system is the most widely used method for prognostic prediction [4]. However, due to substantial molecular heterogeneity, the prognosis for CRC exhibits a considerable variation, even among patients with the same TNM stage [5]. There is an urgent need to explore reliable biomarkers to predict and monitor CRC prognosis [6].

Traditional biomarkers, such as carcinoembryonic antigen (CEA) and carbohydrate antigen 19-9 (CA19-9), have been widely used in clinical practice for predicting the prognosis of CRC patients [7], but they are known to have limited accuracy, with the area under the curve (AUC) of 0.597 and 0.710, respectively [8,9]. Therefore, the development of highly sensitive and specific biomarkers for CRC prognosis is of paramount clinical significance.

High-throughput “omics” techniques, such as metagenomics, proteomics, and metabolomics, represent minimally invasive approaches for biomarker identification and validation [10]. Metabolomics, often positioned downstream of other omics disciplines, allows for precise phenotypic profiling of organisms and their metabolic pathways [11]. In tumor biology, metabolic reprogramming, recognized as one of the hallmarks of cancer, plays a pivotal role in the development and progression of tumors [12]. This process involves significant alterations in the way cancer cells utilize nutrients and generate energy, impacting signaling pathways involved in cell proliferation and invasion, and creating an immunosuppressive tumor microenvironment [13,14]. Moreover, metabolomics offers valuable insights into the interaction between host and gut bacterial metabolites, which is a significant aspect of CRC pathogenesis [15,16]. This paper primarily reports on the recent advancements in the study of metabolomics and its association with CRC prognosis, encompassing its potential in CRC staging, metastasis, recurrence, and survival.

## 2. Metabolomics Profile

Metabolomics, a vital branch of systematic biology, focuses on the study of small molecule metabolites within biological systems, typically referring to molecules with a molecular weight less than 1500 Daltons, including but not limited to glucose, amino acids, nucleotides, lipids, and metabolic byproducts [17,18]. It enables the simultaneous measurement and analysis of hundreds to thousands of chemicals within biological systems [18,19]. Metabolomics can be classified into targeted metabolomics and untargeted metabolomics [20]. Targeted metabolomics involves the analysis of specific metabolites, while untargeted metabolomics can analyze all detectable metabolites in a sample [21]. Metabolomics studies commonly utilize various types of specimens, including blood, urine, feces, and tissue samples [22]. Blood and urine specimens are easily collected and directly reflect an individual’s overall state [23]. Fecal specimens enable the analysis of interactions between host and gut microbial metabolites [24]. Tissue specimens, compared to non-invasive specimens, offer specific advantages, allowing for the analysis of existing metabolite features in tumor tissues [25].

To extract metabolites, plasma or serum samples are generally mixed with acetonitrile, while urine samples are mixed with urease to hydrolyze urea, followed by the addition of methanol. After a process of vortexing, sonication, and incubation, supernatants are isolated via high-speed centrifugation. Tissue samples are immersed in liquid nitrogen and cold methanol. After sonication, the tissue homogenate is mixed with methanol, vortexed, and centrifuged [26]. Fecal samples are homogenized with NaOH, 1-propanol, and pyridine, followed by centrifugation [27].

### 2.1. Detection Platform

Metabolomics technologies mainly include nuclear magnetic resonance (NMR) and mass spectrometry (MS), each with its own advantages and disadvantages [28]. NMR encompasses high-resolution magic angle spinning NMR (HR-MAS NMR), proton nuclear magnetic resonance (^1^H NMR), and magnetic resonance imaging (MRI) [29]. NMR offers excellent reproducibility and high automation and does not require sample pretreatment or derivatization, making it well-suited for high-throughput untargeted metabolomics. However, NMR faces challenges in its relatively low sensitivity and pervasive signal overlap within the NMR spectrum of biological specimens, constraining the identification of metabolites and the revelation of noteworthy biomolecular alterations and biomarkers [30,31]. In comparison, MS provides high sensitivity and specificity and can be coupled with gas chromatography (GC) or liquid chromatography (LC), offering advantages in targeted metabolomics analysis [32]. Gas chromatography–mass spectrometry (GC-MS) includes gas chromatography–time-of-flight mass spectrometry (GC-TOF-MS) and gas chromatography–quadrupole mass spectrometer (GC/Q-MS) [33]. GC-MS offers the advantage of high resolution and sensitivity for analyzing volatile and semi-volatile compounds, using a type of gas as its mobile phase, commonly helium or nitrogen. It has limitations in analyzing thermally labile and polar compounds, and it requires samples to be volatile or to be derivatized prior to analysis [33,34]. Liquid chromatography–mass spectrometry (LC-MS) techniques include conventional high-performance liquid chromatography–quadrupole mass spectrometry (HPLC/Q-MS), ultra-high pressure liquid chromatography–quadrupole mass spectrometry (UPLC/Q-MS), and ultra-high pressure liquid chromatography–quadrupole-time-of-flight tandem mass spectrometry (UPLC/Q-TOF-MS-MS) [35]. LC-MS is a powerful analytical technique for analyzing a wide range of compounds, including polar and non-volatile molecules, without the need for derivatization. The mobile phase typically consists of a solvent or a mixture of solvents, such as methanol, acetonitrile, or their mixture. Nevertheless, it may suffer from longer analysis times, lower resolution for some compounds, and potential ion suppression effects in complex matrices [32,34].

Traditional metabolomics does not provide information on the spatiotemporal distribution of metabolites within biological samples. With the development of imaging mass spectrometry (IMS) technology, spatial metabolomics has emerged, which integrates qualitative and quantitative molecular information with spatiotemporal data for analyzing the qualitative, quantitative, and positional aspects of metabolites in tissues [36]. The IMS technology mainly includes three ionization methods, i.e., matrix-assisted laser desorption/ionization (MALDI), desorption electrospray ionization (DESI), and secondary ion mass spectrometry (SIMS). MALDI is capable of detecting a wide range of molecules in tissue sections, providing precise spatial distribution information. DESI offers the advantage of analyzing solid or semi-solid samples directly from various surfaces under normal pressure conditions without requiring prior sample preparation. SIMS involves the ionization of samples using fast-charged soluble microparticles (e.g., water or acetonitrile) [37].

### 2.2. Data Analysis

#### 2.2.1. Data Preprocessing

Data preprocessing is necessary for metabolomics raw data to provide structured data in an appropriate format before applying statistical analyses. Preprocessing for MS data typically includes baseline correction, alignment of retention time, peak detection and identification, and scoring, while additional spectrum denoising, phase correction, and calibration are needed for NMR [38,39]. Afterward, the spectral information is converted into a dataset in a unified format. Only after undergoing processes such as normalization, scaling, and data transformation can subsequent analyses be carried out [38]. The purpose of normalization is to eliminate any unnecessary errors in the detection process (such as experimental batch effects). Scaling is to improve the results of subsequent multivariate statistical analysis by adjusting the variance structure of the data. Data transformation is to convert the metabolomics data with a skewed distribution into a normal distribution to meet the requirements of linear analysis. It should be noted that metabolomics datasets usually contain missing values. This may be due to biological factors, such as the absence of drug metabolites in non-medicated individuals. It may also be due to limitations of detection techniques, such as low-intensity signals that cannot be separated from the background, signal intensities lower than the detection limit of the instrument, and detection errors caused by unstable instrument performance [40]. Common methods include filling in missing values with zero, half of the minimum detected value (or a specific proportion), or using complex statistical methods such as k-nearest neighbors, a Bayesian model, principal component analysis (PCA), random forest, and multiple imputation by chained equations [41,42]. Recently, deep learning-based methods have been increasingly used to solve the missing values and improve computational accuracy because they can be customized to handle complex missing patterns and data structures [43].

#### 2.2.2. Statistical Analysis

Statistical analyses of metabolomic data can be divided into univariate and multivariate analyses. The univariate analyses, such as *t*-test and rank sum test, are mainly used to examine the differences of each metabolite between different groups. Cox regression is commonly applied to calculate hazard ratios to reflect the association between metabolites and prognosis. When performing univariate analysis on hundreds or thousands of metabolites, multiple hypothesis testing needs to be corrected to control the type I error. Traditional correction methods include Bonferroni and FDR (false discovery rate); however, due to the high correlation between metabolites, these methods are usually too conservative. Alternative methods include the permutation test. By estimating the *p*-value distribution under the null hypothesis, a threshold suitable for sample types and detection methods can be obtained [44]. Since high-dimensional data are generated from metabolomics, multivariate analysis methods are needed to reveal the complicated interrelationships between variables. Commonly used methods include PCA, partial least squares discrimination analysis (PLS-DA), orthogonal PLS-DA, cluster analysis, pathway analysis, enrichment analysis, random forest, and new methods such as LASSO regression and network analysis. In addition, metabolites can be used to construct prediction models, and methods such as C-statistics, NRI (net reclassification improvement), and IDI (integrated discrimination improvement) can be used to evaluate the quality of the model [45].

## 3. Metabolomics and CRC Prognosis

### 3.1. Metabolomics in CRC Staging

CRC prognosis is substantially influenced by the stage at diagnosis. The 5-year survival rate for stage I and II CRC is relatively high, ranging from 87 to 90%, whereas the survival rate is less than 15% for stage IV CRC [46]. A few studies have examined the metabolic features of CRC staging from tissue, plasma, and urine. Wang et al. used ^1^H NMR to distinguish metabolites among the different stages of rectal cancer tissues (stage I = 35, stage II = 37, stage III = 37, and stage IV = 18) and normal controls [47]. They identified a total of 38 differential metabolites, 16 of which were closely correlated with the stage of rectal cancer. However, this study did not report any findings regarding the accuracy, specificity, or sensitivity of these metabolites as potential biomarkers, either individually or in combination, for colorectal cancer. Liu et al. identified eight lipid species in plasma by using the UHPLC-TOF MS method, which can separate early-stage CRC patients (*n* = 20) from advanced-stage CRC (*n* = 20) patients with an AUC of 0.898 [48]. Tian et al. concurrently employed HR-MAS NMR and GC-MS to investigate the metabolic features of CRC tissues, and they discovered significant differences in the fatty acid composition between stage I-II and stage III-IV patients, with an AUC of 0.904 [49].

Liesenfeld et al. used GC-MS and ^1^H-NMR analyses to investigate preoperative (*n* = 97), postoperative 1–8 days (*n* = 12), postoperative 6 months (*n* = 52), and postoperative 12 months (*n* = 38) urine samples from American CRC patients [50]. The results revealed that GC-MS detected two metabolites (including dipeptide of hydroxyproline and p-cresol-β-O-glucuronide) upregulated in stage III-IV patients and downregulated in stage 0-II patients, whereas ^1^H-NMR analysis did not show significant metabolite changes. This study underscores the potential limitations and complexities in metabolite analysis in CRC. Notably, the study performed by Geijsen et al. included the largest number of CRC patients (*n* = 744), and plasma concentrations of 130 metabolites were investigated using LC-MS [51]. They found that compared to stage I-II patients, stage III-IV patients exhibited higher concentrations of sphingolipids and lower concentrations of glycine, histidine, and phosphatidylcholine (Table 1).

These findings indicate the promise of metabolites for the prognosis of CRC. However, the metabolites identified from various studies showed poor overlap, and most of these studies had small sample sizes (<100) and lacked external validation. As a result, it is crucial to conduct further high-quality research, in order to discover and validate reliable biomarkers that can reflect the prognosis of CRC.

### 3.2. Metabolomics in CRC Metastasis

Lymphatic metastasis is the main route of CRC metastasis and plays an important role in CRC prognosis. Thus, it is useful to identify potential metabolomic biomarkers related to the lymph-node metastasis of CRC. Liu, J. et al. collected blood samples from 223 CRC patients from China and analyzed the metabolic levels of amino acids and carnitine in CRC patients with and without lymph-node metastasis by using LC-MS/MS [58]. They revealed that nine serum amino acid metabolites, including leucine, free carnitine, acetylcarnitine, isovalerylcarnitine, glutarylcarnitine, tiglylcarnitine, hexanoylcarnitine, dodecanoylcarnitine, and palmitoylcarnitine exhibited strong predictive capabilities for CRC lymph-node metastasis, with an AUC of 0.947. A study utilized ion mobility–mass spectrometry (IMMS) to probe the tissue metabolites of nine CRC metastatic patients in America [59]. They observed a close association between metabolites and CRC metastasis, including fatty acids, acylcarnitines, bile acids, glucose-1-phosphate, sorbitol, polyamines, and putrescine. Zaimenko et al. examined the plasma metabolites of non-metastasized (*n* = 50) and metachronous metastasized (*n* = 42) CRC patients from Germany using an LC-MS platform [60], and they found that four metabolites (PEG_n=16_, 1,4-D-xylobiose, and two unknown metabolites) showed good performance in predicting metachronous metastasis, with an AUC of 0.820, sensitivity of 0.850, and specificity of 0.770 (Table 2).

The metabolic model of CRC metastasis constructed in the aforementioned study demonstrates high predictive performance. These studies demonstrate the significant relevance of metabolomics in predicting CRC metastasis, with metabolic pathways such as lipid metabolism and amino acid metabolism, which are closely associated with CRC metastasis. However, additional clinical data are needed to analyze and prove the medical value of the identified metabolites in CRC development and metastasis (Table 2).

### 3.3. Metabolomics in CRC Recurrence

In CRC patients, approximately 30-40% of individuals will experience postoperative recurrence. The assessment of postoperative recurrence risk is particularly crucial for enhancing the CRC prognosis. Di et al. utilized NMR to analyze the serum metabolome of 29 CRC patients with recurrence and 65 patients without recurrence [65]. Patients were stratified into high-risk or low-risk groups based on the proximity of metabolomic profiles, and results indicated that the recurrence risk was higher in the high-risk group (50%) than in the low-risk group (17%) (HR = 3.64, 95% CI = 1.50-NA, *p* = 0.0005). Minicozzi et al. used proton magnetic resonance spectroscopy (^1^H MRS) to analyze tissue samples from 29 surgically treated and followed-up CRC patients, revealing that MRS markers (MRS-tm) could differentiate patients with and without recurrence with an AUC of 0.780 [66]. Both studies successfully distinguished CRC patients with recurrence from those without but were limited by a small sample size (Table 3).

Some studies performed analyses among larger populations. Farshidfar et al. used GC-MS to study serum samples from 320 postoperative CRC patients at different stages from Canada, and they constructed a 31-metabolites model to predict CRC recurrence. [67]. Qiu et al. utilized GC-TOF-MS to study tissue samples from 376 CRC patients from China and the United States [68]. They identified 15 metabolites (including significantly elevated β-alanine, glycerol, myristate, palmitoleate, kyrunine, putrescine, cysteine, lactate, glutamate, uracil, hypoxanthine, 5-oxoproline, 2-aminobutyrate, and aspartate, as well as downregulated myo-inositol) that could predict CRC recurrence. Using the predicted probability values for the 5-year recurrence/metastasis rate (R/M) from 15 metabolites, the results demonstrated that patients with lower predicted probability values below the cutoff value had a longer time to recurrence compared to those with higher values above the cutoff (59.2 vs. 25.9 months; *p* = 2.50 × 10^−9^). The AUC was 0.895 (95% CI = 0.824–0.966), sensitivity was 0.750, and specificity was 0.894. This successfully distinguished between CRC patients with favorable and unfavorable prognoses (Table 3).

The studies collectively identified over a hundred metabolites associated with CRC recurrence, including lactate, aspartate, serine, proline, pyruvate, and glutamate, which have been frequently mentioned in the literature. Lactate is one of the most common metabolites associated with alterations in glycolysis, and four studies have indicated a positive association between lactate and CRC recurrence [47,61,64,68]. Serine showed concentration changes in four studies, among which three indicated a positive association between serine concentration and CRC recurrence [47,61,67], while one study reported a negative association [69]. This difference may be attributed to variations in samples and populations.

**Table 3:** Metabolites associated with colorectal cancer recurrence.

Ref.	Specimen	Platform	Population	Positively Associated Metabolites	Negatively Associated Metabolites
Di et al., 2021 [65]	Serum	NMR	Italian	Glutamine, Histidine (HMDB0000177)	Formate (HMDB0304356)
Minicozzi et al., 2013 [66]	Tissue	^1^H-MRS	Italian	Choline (HMDB0000097)	Lipids
Farshidfar et al., 2016 [67]	Serum	GC-MS	Canadian	Aspartic acid (HMDB0000191), Proline, Citric acid (HMDB0000094), Serine (HMDB0000187), Lactate (HMDB0000190)	Ornithine (HMDB0000214), Orotic acid (HMDB0000226), Pyruvic acid (HMDB0000243), Phosphoric acid monomethyl ester, Azelaic acid (HMDB0000784), Erythritol (HMDB0002994), Galactose (HMDB0033704), Erythronic acid (HMDB0000613), Hexadecenoic acid
Qiu et al., 2014 [68]	Tissue	GC-TOF-MS	Chinese; American	β-alanine (HMDB0000056), glycerol (HMDB0000131), myristate, palmitoleate, kyrunine, putrescine (HMDB0001414), cysteine (HMDB0303385), lactate (HMDB0000190), glutamate, uracil (HMDB0000300), hypoxanthine (HMDB0000157), 5-oxoproline, 2-aminobutyrate, aspartate (HMDB0000191)	Myo-inositol (HMDB0000211)
Jonas et al., 2022 [70]	Plasma	Biocrates Absolute IDQ p18	Austrian		PC (HMDB0001565), LPC (HMDB0254271)
Zhuang et al., 2022 [71]	Serum	LC-MS	Chinese	Arginine (HMDB0000517), L-gulono-1,4-lactone, Phenylpyruvate	L-proline, Cis-4-hydroxy-d-proline, Myo-inositol (HMDB0000211), Hippurate
Montcusí et al., 2024 [72]	Plasma	LC-MS	Spanish	Kynurenine/tryptophan	LPC 18:2/PCa 36:2, Hexadecanoylcarnitine
Costantini et al., 2022 [73]	Plasma	^1^H-NMR	Italian	3-hydroxybutyrate, histidine (HMDB0000177), cholesterol (HMDB0000067), triglycerides, phospholipids	

Kynurenine/tryptophan: the ratio of kynurenine to tryptophan.

### 3.4. Metabolomics in CRC Survival

Several studies have analyzed tissue samples from European and American populations. Cai et al. conducted a study using LC-MS to analyze the levels of bile acids in tumor tissues from 228 CRC patients from America [74]. They found that the ratio of glycine-chenodeoxycholic acid to chenodeoxycholic acid was associated with 5-year overall survival (OS) (HR = 3.76, 95% CI = 1.17–12.1, *p* = 0.026), and the ratio of glycine-ursodeoxycholic acid to ursodeoxycholic acid was associated with 5-year recurrence-free survival (RFS) (HR = 3.61, 95% CI = 1.10–11.84, *p* = 0.034). Shen et al. utilized LC-MS to analyze tissue metabolites in 197 CRC patients from America [69]. The results revealed that 11 metabolites (adenosine, asparagine, citrulline, glycerol 3-phosphate (Gro3P), lysophosphatidylcholine (LysoPC) (16:0), ornithine, succinate, threonine, UDP-D-glucose, uracil, and xanthosine) were significantly associated with 5-year OS, while five metabolites (argininosuccinic acid, asparagine, creatinine, hypoxanthine, and serine) were significantly associated with RFS. Additionally, the study demonstrated an association between asparagine and 5-year OS (HR = 6.39, 95% CI = 1.78–22.91) and 5-year RFS (HR = 4.36, 95% CI = 1.39–13.68) in females. Jimenez et al. utilized HR-MAS NMR to investigate tissue samples from 26 CRC surgical resection patients in the United Kingdom [75]. Leveraging 5-year follow-up data, three metabolites (including isobutyrate, acetic acid, and choline) were identified as predictors of 5-year survival in CRC patients and were positively associated with CRC mortality, with an AUC of 0.880. These studies indicate that metabolomics can accurately predict both RFS and OS in CRC patients (Table 4).

Additionally, two studies utilized LC-MS to analyze blood samples from Chinese populations. Wang et al. identified eight plasma metabolites (chenodeoxycholic acid, creatinine, dihydrothymine, histidine-glycine, l-gulonic γ-lactone, l-tryptophan, l-tyrosine, and xanthine) as potential CRC prognostic biomarkers in 34 CRC patients [76]. The results indicated that the low-risk group, defined by eight metabolites, had significantly longer survival compared to the high-risk group. Sun et al. analyzed serum lipid metabolites in 236 CRC patients [77], and they established a CRC prognostic score, named LMS, based on six lipid metabolites, including five high-risk lipid metabolites (Cer (d18:0/14:0), Ganglioside GT3 (d18:0/18:1(9Z), LysoPE (22:6(4Z, 7Z, 10Z, 13Z, 16Z, 19Z)/0:0), PA (20:3(5Z, 8Z, 11Z)/24:1(15Z)), and PS (20:4(5Z, 8Z, 11Z, 14Z)/14:1(9Z)), and one low-risk lipid metabolite (Substance P). Patients with LMS scores greater than or equal to the median LMS value (0.875) were classified into the high-risk group, while the remaining were classified into the low-risk group. The OS in the low-risk group was higher than that in the high-risk group, and the AUC values for predicting the 1-year, 3-year, and 5-year OS were 0.769, 0.711, and 0.723, respectively. Furthermore, the predictive performance improved over 0.800 when integrating clinical factors, albeit with a limited focus on lipid metabolites (Table 4).

The aforementioned study indicated the predictive performance of metabolites on the RFS, PFS, and OS of CRC. Survival-associated metabolites identified encompassed lyso-phosphatidylcholine, bile acids, lipids, tyrosine, xanthine, and asparagine, among others. These metabolites are typically involved in bile acid, amino acid, and purine metabolism pathways. However, a clear understanding of the molecular mechanisms underlying the effects of these metabolites on tumorigenesis is lacking. Further research utilizing cellular and animal models is needed to elucidate the molecular mechanisms.

## 4. Conclusions and Future Directions

The metabolic regulation of tumor cells stands as a critical determinant for tumor progression and survival [86]. Metabolomic investigations into CRC prognosis offer pivotal insights into the development of novel prognostic tumor markers in CRC. Currently, researchers primarily employ NMR, LC-MS, CE-TOFMS, and IMMS to analyze the changes and patterns of endogenous metabolic biomarkers occurring in the staging, metastasis, recurrence, and survival outcomes of CRC patients. Metabolites that undergo alterations in CRC prognosis mainly include sugars, lipids, and amino acids. Sugars play a critical role in the energy metabolism of tumor cells, which typically exhibit increased glucose uptake and utilization, a phenomenon known as the Warburg effect. Through this mechanism, tumor cells can more efficiently acquire the energy and raw materials required for biosynthesis [87]. Tumor cells also exhibit a high demand for lipids, particularly for membrane biosynthesis and cell signaling. Abnormalities in lipid metabolism may lead to membrane instability and abnormal activation of signaling pathways, thereby affecting cancer cell proliferation and invasion [88]. Aberrant amino acid metabolism is associated with tumor growth and metastasis; the metabolic products of amino acids can influence cell apoptosis and oxidative stress responses, thereby impacting the biological behavior of tumors [89].

Multiple metabolites have been consistently identified to be associated with CRC prognosis in different studies, such as lactate, which is involved in glycolysis; and amino acid metabolite products alanine, arginine, and proline, as well as taurine, which are involved in gut microbiota metabolism. However, many metabolites, such as glutamate, creatinine, and ornithine, have shown inconsistent results. Previous studies exhibited variations in sample size, criteria for participant inclusion and exclusion, metabolomic platforms utilized, and analytical methods employed, potentially accounting for the observed inconsistencies.

Metabolomics, as an ideal biomarker detection technology, can assist in predicting CRC prognosis at the molecular level. However, translating these findings into clinical practice and personalized medicine poses several challenges. The selection of sample types should be assessed based on different clinical applications. While previous studies have included blood, tissue, urine, and fecal samples, relatively fewer efforts have been made to investigate different sample types or their combinations to determine the most suitable sample for different clinical applications. Some studies lack sensitivity and specificity data for the metabolic biomarkers, and certain biomarkers (including cysteine, glutathione, serine, succinic acid, triglycerides, tyrosine, etc.) (Table 2) exhibit conflicting trends across different studies. The replication of current findings requires further large-scale studies stratified by different phenotypes. Despite the close association between metabolic biomarkers and CRC prognosis, the molecular mechanisms underlying this association remain unclear. Further research in cellular and animal models is needed to elucidate their molecular mechanisms. To further enhance the prognostic models for CRC, integration of metabolomics with other omics approaches, such as metagenomics, proteomics, etc., could be considered to advance our understanding of the systems biology of CRC.

With the advancement of high-throughput genomic sequencing technologies, numerous genetic mutations have been implicated in the prognosis of CRC patients [90]. Notably, some mutations in specific genes and pathways, such as microsatellite instability [91], APC, TP53, KRAS, BRAF, WNT signaling, and TGF-β/SMAD2/3 signaling [92,93,94,95,96,97], have emerged as prognostic biomarkers for CRC.

CEA is a specific blood glycoprotein biomarker approved by the US FDA for detecting CRC recurrence [98]. Furthermore, proteomics studies have revealed potential prognostic biomarkers for CRC, such as S100A9 in serum [99], collagen type XII in urine [100], and STK4 in tissue [101].

The biological mechanism of CRC is highly complex, necessitating a multi-omics approach for a comprehensive understanding [102]. The workflows involved in multi-omics integration analysis encompass data generation via high-throughput omics platforms, preprocessing and dimensionality reduction using principal component analysis or gene co-expression network analysis, and prediction analysis through statistical methods like Bayesian and Markov models [103,104,105,106].

In conclusion, metabolomics has emerged as an important approach for identifying biomarkers related to CRC prognosis. However, current research confronts challenges such as technical variability, biological heterogeneity, and the need for large-scale studies. Future investigations will necessitate further exploration into integrating metabolomics with other omics disciplines, such as genomics and proteomics, to provide a more comprehensive understanding of CRC biology and identify novel prognostic markers.

## Figures and Tables

**Table 1:** Metabolites associated with colorectal cancer staging.

Ref.	Specimen	Platform	Population	Positively Associated Metabolites	Negatively Associated Metabolites
Wang et al., 2013 [47]	Tissue	^1^H-NMR	Chinese	Lactate (HMDB0000190), L-threonine (HMDB0000167), Acetate, Glutathione (HMDB0000125), Uracil (HMDB0000300), Succinate, Serine (HMDB0000187), fFormate (HMDB0304356), Lysine (HMDB0000182), Tyrosine (HMDB0000158)	Myo-inositol (HMDB0000211), Taurine (HMDB0000251), Phosphocreatine (HMDB0001511), Creatine (HMDB0000064), Betaine (HMDB0000043), Dimethylglycine (HMDB0000092)
Liu et al., 2019 [48]	Plasma	LC-MS	Chinese	CE (20:4), TAG	FAHFA 27:1
Tian et al., 2016 [49]	Tissue	GC-MS; HR-MAS-NMR	Chinese	Lipid	Choline (HMDB0000097), PC (HMDB0001565), GPC (HMDB0000086), PE (HMDB0060244), Scyllo-inositol (HMDB0006088), Glutathione (HMDB0000125), Taurine (HMDB0000251), Uracil (HMDB0000300), Isocytosine, Inosine (HMDB0000195), Glutamine, Glutamate, Aspartate (HMDB0000191), Asparagine (HMDB0000168), Glycine (HMDB0000123), Cysteine (HMDB0303385)
Liesenfeld et al., 2015 [50]	Urine	GC-MS; ^1^H-NMR	American	Dipeptide of hydroxyproline, P-cresol-β-O-glucuronide	
Geijsen et al., 2020 [51]	Plasma	LC-MS	Dutch; German; Austrian	Sphingolipids	Glycine (HMDB0000123), Hhistidine (HMDB0000177), Phosphatidylcholine
Mirnezami et al., 2014 [52]	Tissue	HR-MAS-NMR	British	Triglycerides, Acetate	GPC (HMDB0000086)
Zheng et al., 2022 [53]	Tissue	iEESI-MS	Chinese	Hypoxanthine (HMDB0000157), LPC (HMDB0254271), Glucose (HMDB0000122), PE (HMDB0060244), SM, L-Palmitoylcarnitine (HMDB0240774), PC (HMDB0001565)	Cholesterol sulfate (HMDB0000653), Glycerophosphoinositol (HMDB0011649), PG (HMDB0302468), Inosine (HMDB0000195), Inositol cyclic phosphate (HMDB0001125), Taurine (HMDB0000251), Palmitoleic acid (HMDB0003229)
Coradduzza et al., 2022 [54]	Plasma	LC-MS	Italian	Agmatine (HMDB0001432), Arginine (HMDB0000517), Cadaverine (HMDB0002322), Lysine (HMDB0000182), Ornithine (HMDB0000214), Putrescine (HMDB0001414), Acetyl-putrescine, Spermine (HMDB0001256), Acetyl-spermine	
Kang et al., 2023 [55]	Tissue	LC-MS	Chinese	2-Aminobenzenesulfonic acid (HMDB0304940), P-sulfanilic acid, Quinoline-4-carboxylic acid (HMDB0257047), Methylcysteine (HMDB0002108), 5′-Deoxy-5′-(methylthio) adenosine	N-ɑ-acetyl-ε-(2-propenal)-Lys
Zhang et al., 2024 [56]	Plasma	LC-MS	American	Aminoadipate (HMDB0000510), Lysine (HMDB0000182), L-glutamic acid, Choline (HMDB0000097), 2-aminomuconic acid (HMDB0001241), Tyrosine (HMDB0000158)	Nicotinamide, Acetyl-l-carnitine, L-threonine (HMDB0000167), 4,6-quinolinediol, 2-aminosuccinamate, Uridine (HMDB0000296), Urea (HMDB0000294), Cytosine (HMDB0000630), Uracil (HMDB0000300)
Ishizaki et al., 2024 [57]	Plasma	CE-TOF-MS	Japanese	N^1^-acetylspermine, N^1^, N^12^-diacetylspermine, Spermine (HMDB0001256), Spermidine (HMDB0001257)	Histidine (HMDB0000177), O-acetylcarnitine

GPC: glycerophosphorylcholine, PC: phosphorylcholine, PE: phosphoethanolamine, CE: cholesteryl ester, TAG: triacylglyceride, FAHFA: fatty acyl esters of hydroxy fatty acid, LPC: lysophosphatidylcholine, SM: sphingomyelin, PG: phosphatidylglycerol.

**Table 2:** Metabolites associated with colorectal cancer metastasis.

Ref.	Specimen	Platform	Population	Positively Associated Metabolites	Negatively Associated Metabolites
Liu et al., 2020 [58]	Serum	LC-MS	Chinese	Leucine (HMDB0000687), Free carnitine, Acetylcarnitine (HMDB0000201), Isovalerylcarnitine (HMDB0000688), Glutarylcarnitine (HMDB0013130), Tiglylcarnitine (HMDB0002366), Dexanoylcarnitine (HMDB0000756), Dodecanoylcarnitine (HMDB0002250), Palmitoylcarnitine (HMDB0000222)	
Williams et al., 2015 [59]	Tissue	IMMS	American	3-Hydroxysuberic acid (HMDB0000325), Docosapentaenoic acid, 3-Oxooctadecanoic acid (HMDB0010736), 3,4-Dihydroxyphenylvaleric acid (HMDB0029233), Acylcarnitines, Bile acids, Glucose-1-phosphate (HMDB0001586), Sorbitol (HMDB0000247), Polyamines, Putrescine (HMDB0001414)	
Zaimenko et al., 2019 [60]	Plasma	LC-MS	German	1,4-D-xylobiose	PEG_n=16_
Zhang et al., 2016 [61]	Tissue	^1^H-NMR	Chinese	Lactate (HMDB0000190), L-threonine (HMDB0000167), Lipids, Succinate, Dimethylglycine (HMDB0000092), Serine (HMDB0000187), Arginine (HMDB0000517), Uracil (HMDB0000300)	Glucose (HMDB0000122), Ketoglutarate, Phosphocreatine (HMDB0001511), Creatine (HMDB0000064), Myo-inositol (HMDB0000211)
Zhang et al., 2020 [62]	Serum	LC-MS	Chinese	Tyramine (HMDB0000306), Abscisic acid, (HMDB0036093) 3-hydroxynonanoyl carnitine (HMDB0061635), Ethanolamine oleate, Coutaric acid (HMDB0029225), Calcitroic acid (HMDB0006472), Lithocholic acid (HMDB0000761), Treprostinil (HMDB0014518), Flavoxate (HMDB0015279), Glycine conjugate, Glucosylsphingosine (HMDB0000596)	Sorgoleone, Aldosterone (HMDB0000037), Cinncassiol C3 (HMDB0036859), hydroxy-5-(3′, 5′-dihydroxyphenyl)-valeric acid-O-glucuronide, Phenobarbital O-glucuronide, Pinostrobin 5-glucoside
Elmallah et al., 2022 [63]	Plasma	LC-MS	American	Cer	PC (HMDB0001565), PE (HMDB0060244)
Tristán et al., 2023 [64]	Serum	NMR	Spanish	Lactate (HMDB0000190), Glutamate, Pyruvate, Acetate, Acetone (HMDB0001659)	3-hydroxybutyrate, Glutamine, Alanine (HMDB0000161), Isoleucine (HMDB0000172), Valine (HMDB0000883), Choline (HMDB0000097), GPC (HMDB0000086)

PEG: polyethylene glycol, Cer: ceramide.

**Table 4:** Metabolites associated with colorectal cancer survival.

Ref.	Specimen	Platform	Population	Positively Associated Metabolites	Negatively Associated Metabolites
Shen et al., 2022 [69]	Tissue	HILIC-MS; RPLC-MS	American	Asparagine (HMDB0000168), Citrulline (HMDB0000904), Glycerol 3-phosphate (HMDB0000126), LPC (16:0), Uracil (HMDB0000300), Xanthosine (HMDB0000299)	Adenosine, Arginino, Succinic acid (HMDB0000254), Hypoxanthine (HMDB0000157), Serine (HMDB0000187), Succinate, L-threonine (HMDB0000167), UDP-D-Glucose
Cai et al., 2022 [74]	Tissue	LC-MS	American	Glycine-chenodeoxycholic acid/Chenodeoxycholic acid, Glycine-ursodeoxycholic acid/Ursodeoxycholic acid	
Jiménez et al., 2013 [75]	Tissue	^1^H-HR-MAS-NMR	British	Isobutyrate, Acetic acid (HMDB0000042), Choline (HMDB0000097)	
Wang et al., 2019 [76]	Plasma	LC-MS	Chinese	Dihydrothymine l-gulonic γ-lactone	Chenodeoxycholic acid (HMDB0000518), Creatinine (HMDB0000562), Histidine-glycine, L-tryptophan, Tyrosine (HMDB0000158), Xanthine (HMDB0000292)
Sun et al., 2022 [77]	Serum	LC-MS	Chinese	Cer (d18:0/14:0), Ganglioside GT3 (d18:0/18:1(9Z), LPE (22:6(4Z, 7Z, 10Z, 13Z, 16Z, 19Z)/0:0), PA (20:3(5Z, 8Z, 11Z)/24:1(15Z)), PS (20:4(5Z, 8Z, 11Z, 14Z)/14:1(9Z	Substance P
Figueiredo et al. 2018 [78]	Serum	MALDI-TOF-MS	Brazilian		Sphingolipids, Policetidios, Glycerophospholipid
Ecker et al., 2021 [79]	Tissue	ESI-MS/MS	German	SM, TG	Cer
Sakurai et al., 2022 [80]	Urine	CE-TOF-MS	Japanese	γ-Guanidinobutyrate	
Yang et al., 2022 [81]	Plasma	LC-MS		PC (HMDB0001565), LPC (HMDB0254271)	TG
Xie et al., 2023 [82]	Fecal	LC-MS	Chinese		N-acetylmannosamine (HMDB0001129), 2,5-dihydroxybenzaldehyde
Ose et al., 2023 [83]	Plasma	Biocrates Absolute IDQ p180	Dutch; German; Austrian	Proline, Hexose, Propionylcarnitine (HMDB0000824), Sarcosine (HMDB0000271), Hydroxybutyrylcarnitine, t4-hydroxyproline, Creatinine (HMDB0000562), Symmetric Dimethylarginine (HMDB0003334), Decenoylcarnitine (HMDB0250918), Asymmetric dimethylarginine (HMDB0001539)	Histidine (HMDB0000177), SM, PC (HMDB0001565), LPC (HMDB0254271), Octadecanoylcarnitine, Hydroxysphingomyeline
Damerell et al., 2024 [84]	Plasma; Serum	LC-MS	Dutch; German; Austrian; American	3-hydroxykynurenine, Quinolinic acid (HMDB0000232), Kynurenine (HMDB0000684)	Tryptophan, Xanthurenic acid (HMDB0000881), Picolinic acid (HMDB0002243)
Jain et al., 2024 [85]	Tissue	LC-MS	American	Ethyl-4-aminobenzoate, Theobromine (HMDB0002825), Prostaglandine E2, Kynurenic acid (HMDB0000715), Riboflavin (HMDB0000244), Glycylproline (HMDB0000721), Hydrocinnamic acid (HMDB0000764), N-Acetylleucine, LPC (20:3) (HMDB0010393), P-Toluenesulfonic acid (HMDB0059933), Cytidine (HMDB0000089) , Linolenic Acid, Ethyl laurate, N-Acetyl-L-aspartic acid, Palmitoleic acid (HMDB0003229), Turanose (HMDB0011740), Indirubin (HMDB0240743), Retinyl acetate (HMDB0003648), Thiamine pyrophosphate (HMDB0001372), Uracil (HMDB0000300)	4-methyl-2-oxovalerate, Methionine (HMDB0000696), Tyrosine (HMDB0000158), S-Adenosyl-methionine, N-Acetyl-L-methionine, ilirubin, Alanyl-L-phenylalanine, Threoninyl-Leucine (HMDB0029065), Valyl-Methionine (HMDB0029133), Valyl-Serine (HMDB0029136), Ergothioneine (HMDB0003045), LPC (14:0), LPE (P18:0) (HMDB0011130), 3-Hexenedioic acid (HMDB0000393), Phenylacetylglycine (HMDB0000821), Pantothenate, Hexanoylcarnitine (HMDB0000756), Guanosine (HMDB0000133), 8-Hydroxy-2′-deoxyguanosine, Arachidonic acid (HMDB0001043), N2-gamma-glutamylglutamine, Glucose-6-phosphate (HMDB0001401), Butyrylcarnitine (HMDB0002013), CMP sialic acid, Glycyl-L-phenylalanine, Inosine 5 triphosphate, Alanine (HMDB0000161), Cystine glutathione, LPC (20:4), Myristoylcarnitine (HMDB0254979), N-a-Acetyl-glutamine, Methylaspartic acid, Oleoylcarnitine (HMDB0005065), Uric acid (HMDB0000289), Valerylcarnitine (HMDB0013128), Valyl aspartate, Kynurenine (HMDB0000684), Creatinine (HMDB0000562), Trans-Urocanic acid, 5-Aminovaleric acid, 2-Deoxyguanosine, Alanylglutamic acid (HMDB0028686), dAMP, Estradiol-17B-glucuronide, Glycyl-L-glutamine, Glycyl-Serine (HMDB0028850), N2-Acetylornithine (HMDB0003357), Decanoylcarnitine (HMDB0000651), Adenosine 5′-diphosphate

LPE: lysophosphatidylethanolamine, PA: diacylglycerophosphate, PS: phosphatidylserine, TG: triacylglycerol, CMP: cytidine 3′-monophosphate, AMP: Adenosine monophosphate, Glycine-chenodeoxycholic acid/Chenodeoxycholic acid: the ratio of glycine-chenodeoxycholic acid to chenodeoxycholic acid, Glycine-ursodeoxycholic acid/Ursodeoxycholic acid: the ratio of glycine-ursodeoxycholic acid to ursodeoxycholic acid.

## Data Availability

No new data were created or analyzed in this study.
